# Assessment of mitral valve regurgitation by cardiovascular magnetic resonance imaging

**DOI:** 10.1038/s41569-019-0305-z

**Published:** 2019-12-09

**Authors:** Pankaj Garg, Andrew J. Swift, Liang Zhong, Carl-Johan Carlhäll, Tino Ebbers, Jos Westenberg, Michael D. Hope, Chiara Bucciarelli-Ducci, Jeroen J. Bax, Saul G. Myerson

**Affiliations:** 1 Department of Infection, Immunity and Cardiovascular Disease, University of Sheffield, Sheffield, UK; 2 National Heart Centre Singapore, Duke–NUS Medical School, National University of Singapore, Singapore, Singapore; 3 Department of Medical and Health Sciences, Linköping University, Linköping, Sweden; 4 Department of Radiology, Leiden University Medical Center, Leiden, Netherlands; 5 Department of Radiology, University of California-San Francisco, San Francisco, CA USA; 6 Bristol Heart Institute, Bristol National Institute of Health Research (NIHR) Biomedical Research Centre, University Hospitals Bristol NHS Trust and University of Bristol, Bristol, UK; 7 Department of Cardiology, Leiden University Medical Center, Leiden, Netherlands; 8 Departments of Cardiology and Cardiovascular Medicine, University of Oxford Centre for Clinical Magnetic Resonance Research, John Radcliffe Hospital, Oxford, UK

**Keywords:** Valvular disease, Magnetic resonance imaging, Echocardiography, Diagnosis

## Abstract

Mitral regurgitation (MR) is a common valvular heart disease and is the second most frequent indication for heart valve surgery in Western countries. Echocardiography is the recommended first-line test for the assessment of valvular heart disease, but cardiovascular magnetic resonance imaging (CMR) provides complementary information, especially for assessing MR severity and to plan the timing of intervention. As new CMR techniques for the assessment of MR have arisen, standardizing CMR protocols for research and clinical studies has become important in order to optimize diagnostic utility and support the wider use of CMR for the clinical assessment of MR. In this Consensus Statement, we provide a detailed description of the current evidence on the use of CMR for MR assessment, highlight its current clinical utility, and recommend a standardized CMR protocol and report for MR assessment.

## Abstract (web summary)

In this Consensus Statement, Garg and colleagues describe the current evidence on the use of cardiovascular magnetic resonance imaging for the assessment of mitral regurgitation, highlight its current clinical utility, and recommend a standardized imaging protocol and report.

## Introduction

Mitral regurgitation (MR) is a common valvular heart disease and is the second most frequent indication for heart valve surgery in Western countries^1^. Almost 9% of the general population aged >75 years in the USA have MR^2^. Cardiac imaging is crucial for diagnosis, identifying the cause of the disease, monitoring disease progression and planning definitive treatment for MR^3^. Echocardiography remains the first-line and most widely available imaging test for the assessment of MR. Cardiovascular magnetic resonance imaging (CMR) has also emerged in the past 20 years as a robust, noninvasive imaging modality for the assessment of patients with MR^4^. CMR offers a comprehensive evaluation of MR and its effects on the heart by providing precise volumetric assessment (using cine images) and myocardial scar or fibrosis assessment (using the late gadolinium enhancement (LGE) technique). Therefore, CMR is often complementary to echocardiography in informing the clinical management of MR^4^.

As more evidence is gathered for the use of CMR in the assessment of MR, clinicians and researchers need a standard CMR protocol to follow in order to improve the consistency of mitral valve assessment. A consistent approach will further improve the clinical translation and adoption of CMR for the assessment of the mitral valve and MR. Although evidence-based data from randomized clinical trials are limited for MR assessment, in this Consensus Statement we highlight the role of CMR in the assessment of MR and provide recommendations for a standardized protocol and reporting method.

## Methods

A panel of individuals with vast expertise in MR assessment by standard and emerging methods of CMR gathered in a closed group meeting titled ‘Mitral Valve Regurgitation Assessment by Cardiac Magnetic Resonance’ held at the joint EuroCMR–Society for Cardiovascular Magnetic Resonance (SCMR) meeting in Barcelona, Spain, in January 2018. The organizers of the meeting (P.G. and J.W.) appointed a scientific committee (A.J.S., L.Z., C.-J.C. and T.E.) to discuss the appropriateness of the meeting, clinical needs, topics to be discussed and, more importantly, to identify experts in the field to cover all aspects relevant to the goal of the meeting. The appointment of international experts was decided by general agreement among the members of the scientific committee. A follow-up meeting was arranged at the Joint Annual Meeting ISMRM–ESMRMB in Paris, France, in June 2018. A final teleconference was arranged in February 2019, after completion of an electronic database search and collection of evidence.

The evidence-based literature was synthesized by the following authors: P.G., A.J.S., L.Z. and C.-J.C. They searched electronic databases including MEDLINE (PubMed), Embase, the Cochrane library and OpenGray up to 8 January 2019, with no restriction on language. The search terms included “mitral regurgitation”, “cardiovascular magnetic resonance” and “echocardiography”. All retrospective and prospective studies that reported both CMR and echocardiography of MR were considered eligible. In addition, A.J.S. checked the reference lists of selected articles for further relevant articles. Review articles, case reports, comments and author replies were excluded. The final decision on inclusion was reached through a consensus of the four screening authors.

The main objectives at the meetings were to appraise previous and new lines of evidence on CMR-based assessment of the mitral valve, to review available data on the diagnostic and prognostic value of CMR in the MR setting, and to provide recommendations for the standardization of imaging protocols for use in clinical trials and experimental scenarios. For the consensus recommendations, open discussions took place between all experts, and verbal agreements were made. A majority of experts had to agree with a statement or recommendation for it to be included in this Consensus Statement. This Consensus Statement summarizes the final conclusions and recommendations agreed by the expert panel in the meetings.

## Current clinical guidelines

The ESC and AHA/ACC guidelines for the management of valvular heart disease emphasize the severity of the MR in deciding whether patients are eligible for mitral valve surgery^3,5^, while also emphasizing the importance of assessing the haemodynamic effects of the MR on the left ventricle and left atrium. The AHA/ACC guidelines highlight that CMR is an appropriate test in chronic primary MR to assess ventricular volumes and function or even MR severity, especially when these issues are not satisfactorily addressed by transthoracic echocardiography (TTE)^5^. In addition, for chronic secondary MR, CMR is indicated to establish and/or to assess myocardial viability, which in turn might influence the management of functional MR^5^. Similarly, the ESC guidelines on valvular heart disease recommend CMR assessment in patients with inadequate TTE imaging for ventricular volume and function assessment^3^. These guidelines do not detail several additional areas in which CMR can provide information on the aetiology of MR (primary or secondary), including the assessment of mitral valve leaflet or scallop function^6^. Moreover, the guidelines have limited recommendations on how to perform comprehensive assessment of MR by CMR in a standardized way.

## Evidence for CMR to assess MR

CMR is an emerging, noninvasive tool that can provide comprehensive assessment of the mitral valve and MR. As previously stated, CMR provides excellent accuracy and reproducibility in the assessment of ventricular and atrial size and function^7^, allowing for comprehensive longitudinal and postoperative assessment of reverse left ventricular (LV) remodelling. Studies evaluating the role of CMR for the assessment of MR are listed in Table 1. A thorough evaluation with the use of cine CMR allows a systematic inspection of the anatomy of the mitral valve and characterizes the MR, both of which contribute to determining the aetiology of the MR^8^. The severity of the MR can be evaluated using several CMR-based quantitative techniques that are detailed below. Furthermore, CMR can provide information about the mechanism of MR by identifying morphological abnormalities of the mitral valve apparatus^9,10^. The presence of billowing or flail segments can be identified by dedicated cine imaging focusing on the different scallops of the mitral valve leaflets^8^. In secondary MR, CMR can provide an accurate assessment of LV dilatation and (dys)function, in addition to the identification of myocardial and papillary muscle scar formation^11^.

**Table 1:** Studies assessing the use of CMR in MR with or without echocardiography

Study (year)	*n*	Prospective study?	Correlation (*r*)^a^	Bias (ml)^b^
***LVSV – AoPC method***				
Penicka et al. (2018)^13^	258	Yes	0.61	17.1 ± 28.9
Heo et al. (2017)^44^	37	Yes	PISA: 0.81	–15.2 ± 18.3
			2D volumetric: 0.56	–17.4 ± 29.4
			3D echo: 0.94	8.7 ± 11.6
Harris et al. (2017)^45^	22	Yes	–	–
Sachdev et al. (2017)^46^	50	Yes	0.79	–0.6 (–43 to 44)
Myerson et al. (2016)^12^	109	Yes	–	–
Aplin et al. (2016)^47^	72	Yes	0.80	11 ± 28
Lopez-Mattei et al. (2016)^48^	70	No	0.59	2 ± 17
Uretsky et al. (2015)^19^	103	Yes	0.60	16 (–38 to 70)
Brugger et al. (2015)^49^	55	Yes	3D TOE PISA: 0.87	–5.9 (–26.5 to 14.7)
			3D TTE PISA: 0.74	–11.8 (–39.4 to 15.8)
Choi et al. (2014)^50^	52	Yes	2D TTE PISA: 0.84	–10.4 (–29.8 to 8.9)
			3D TTE PISA: 0.91	0.9 (–12.8 to 14.7)
Van De Heyning et al. (2013)^51^	38	Yes	2D TTE Doppler: –0.14	39 (limits not reported)
			TTE PISA: 0.45	30 (limits not reported)
Thavendiranathan et al. (2013)^52^	35	Yes	3D integrated PISA: 0.92	1.4 (–17 to 19.8)
			3D peak PISA: 0.87	15.3 (–10.2 to 40.8)
Son et al. (2013)^53^	32	Yes	2D PISA: 0.55	7.9 (–46.9 to 62.8)
			2D VM: 0.58	16.7 (–44.9 to 78.2)
			3D FVCD: 0.85	5.7 (–27.9 to 39.3)
Reddy et al. (2013)^54^	44	Yes	–	–
Cawley et al. (2013)^55^	10	Yes	PISA: 0.96	–4 (–29 to 22)
			Doppler: 0.85	21 (–28 to 72)
Hamada et al. (2012)^56^	46	Yes	EROA: 0.75	20 (–41 to 81)
			AROA: –	13 (–22 to 47)
Skaug et al. (2010)^57^	27	Yes	0.78	–4.7 ± 30.6
Shanks et al. (2010)^43^	30	Yes	2D TTE: –	–12.4 (–45.6 to 20.8)
			3D TEE: –	–2.32 (–18.6 to 13.9)
Myerson et al. (2010)^58^	55	Yes	–	–
Hellgren et al. (2008)^59^	18	Yes	–	–27.5 (–65.4 to 10.3)
Gabriel et al. (2008)^60^	27	Yes	–	–
Gelfand et al. (2006)^61^	107	Yes	–	–
Kizilbash et al. (1998)^62^	22	Yes	0.92	3 ± 13
Hundley et al. (1995)^63^	17	Yes	–	–
***LVSV – RVSV method***				
Sukpraphrute et al. (2012)^31^	43	No	PISA: 0.48	–6.4 (–49 to 36)
Kon et al. (2004)^35^	28	No	–	–
***MVPC – AoPC method***				
Polte et al. (2017)^64^	40	Yes	–	–
Buck et al. (2008)^65^	73	Yes	0.63	–13.5 ± 10.3
Fujita et al. (1994)^66^	19	Yes	–	–
***4D-flow methods***				
Kamphuis et al. (2019)^67^	160	No	–	–
Feneis et al. (2018)^30^	21	No	Direct: 0.81	–
			Indirect: 0.97	–
Calkoen et al. (2015)^37^	32	Yes	0.50–0.60	–
Roes et al. (2009)^28^	51	No	–	–
Marsan et al. (2009)^68^	64	Yes	3D TTE: 0.94	–0.08 (–7.7 to 7.6)
			2D TTE: –	–2.9 (–18 to 12.5)
Westenberg et al. (2008)^38^	30	No	–	–
***Other quantitative methods***				
Gorodisky et al. (2018)^69^	27	Yes	CMR PISA versus echo PISA: 0.87	–
Uretsky et al. (2010)^70^	23	No	–	–
***Nonquantitative methods***				
Heitner et al. (2012)^71^	68	No	0.47	–
Ozdogan et al. (2009)^72^	21	No	–	–
Buchner et al. (2008)^34^	35	Yes	CMR EROA versus echo EROA: 0.81	–
Aurigemma et al. (1990)^73^	50	Yes	–	–
Pflugfelder et al. (1989)^74^	26	Yes	–	–

AoPC, aortic phase-contrast stroke volume; AROA, anatomical regurgitant orifice area; CMR, cardiac magnetic resonance imaging; echo, echocardiography; EROA, effective regurgitant orifice area; FVCD, full-volume colour Doppler echocardiography; LVSV, left ventricular stroke volume; MR, mitral regurgitation; MVPC, mitral valve phase-contrast stroke volume; PISA, proximal isovelocity surface area; RVSV, right ventricular stroke volume; TOE, transoesophageal echocardiography; TTE, transthoracic echocardiography; VM, volumetric quantification method. ^a^Between echocardiography-determined and CMR-determined MR volume. ^b^Regurgitant volume (echocardiography – CMR).

### Clinical outcome studies

#### Primary MR

In a prospective, multicentre study by Myerson and colleagues, 109 asymptomatic patients with moderate or severe primary MR defined by echocardiography had CMR scans at baseline and were followed up for up to 8 years (mean 2.5 ± 1.9 years)^12^. CMR quantification of MR accurately identified patients who progressed to having symptoms or other indications for surgery: 91% of participants with a regurgitant volume ≤55 ml survived to 5 years without surgery compared with only 21% of participants with a regurgitant volume >55 ml (*P* < 0.0001)^12^. Similar findings were observed in a further prospective, dual-centre study by Penicka and colleagues in which 258 asymptomatic patients with moderate or severe primary MR defined by echocardiography underwent CMR and were followed up for a median of 5 years^13^. In this study, the researchers demonstrated that CMR-derived MR volume was the best predictor of mortality (area under the curve = 0.72). In addition, when MR volume was combined with the development of an indication for mitral valve surgery as a clinical outcome, the predictive value increased (area under the curve = 0.83)^13^. The other major finding of this study was that the agreement between echocardiography and CMR for classifying primary MR was poor for patients with late systolic MR or multiple MR jets (both *κ* < 0.2)^13^. These findings suggest that, in patients who have complex primary MR jet physiology, standard CMR quantification of MR can offer complementary information to that obtained by echocardiography for consideration of valvular intervention.

LGE has been reported on CMR images in patients with primary MR, especially in those with mitral valve prolapse^8,14^. In addition, in patients with primary MR, LGE of papillary muscles is associated with complex ventricular arrhythmias^15^. Subsequent evidence confirms that LV fibrosis indicated by LGE is more prevalent in MR with mitral valve prolapse than in patients without prolapse, whereas patients with mitral valve prolapse and concomitant LV fibrosis have the highest rate of arrhythmic events^15^. Persistent volume overload from MR results in impaired LV function and the presence of diffuse myocardial fibrosis^16^. Mitral annulus disjunction is an abnormal atrial displacement of the hinge point of the mitral valve away from the ventricular myocardium^17^. Mitral annulus disjunction has been associated with mitral valve prolapse and sudden cardiac death owing to ventricular arrhythmias^9,18^.

#### Secondary MR

A prospective, multicentre study in 103 patients with either primary or secondary MR showed substantial discordance in the severity of MR as assessed with either CMR or echocardiography on the basis of either the American Society of Echocardiography integrated method or the proximal isovelocity surface area (PISA)-based regurgitant volume^19^. In addition, in the subset of patients who had mitral valve surgery and underwent postoperative CMR, good correlation existed between LV remodelling and MR severity as assessed by CMR (*r* = 0.85; *P* < 0.0001), but not when assessed by echocardiography (*r* = 0.32; *P* = 0.1), either categorically or quantitatively, with the use of the PISA method.

Persistent volume overload from MR results in impaired LV function and subsequent myocardial fibrosis^16^. In patients with ischaemic cardiomyopathy and severe MR, the presence of severe scarring in the region of the posterior papillary muscle, as detected by preoperative CMR, can render these patients unsuitable for mitral annuloplasty^20^. Moreover, the extent of myocardial scarring is also informative about the progression of ischaemic MR^21^.

In summary, CMR has become an established noninvasive imaging modality to assess the severity of MR. CMR can be used to phenotype prognostically relevant clinical features that are complementary to those identified by echocardiography in patients with MR.

## CMR acquisition protocol to assess MR

**Fig. 1:**
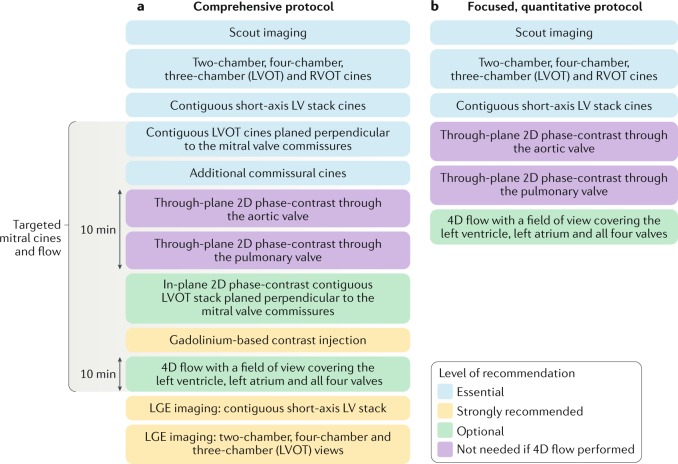
Recommended cardiovascular magnetic resonance imaging protocols for the assessment of mitral regurgitation. **a** | Comprehensive cardiovascular magnetic resonance imaging protocol for the assessment of mitral regurgitation. **b** | Focused, quantitative protocol. LGE, late gadolinium enhancement; LV, left ventricular; LVOT, left ventricular outflow tract; RVOT, right ventricular outflow tract.

A CMR study should aim to answer several clinical questions (Box 1) that influence the management of patients with MR. A comprehensive MR protocol should assess the mitral valve anatomy and function to define the cause of the MR — primary (organic) versus secondary (functional), LV and right ventricular (RV) volumes and function, and quantify the MR (Fig. 1a). However, given that TTE, the first-line imaging test, can provide information on the aetiology of the MR in the majority of patients, we also include a CMR protocol focused on MR quantification (Fig. 1b). A decision on whether to use the comprehensive or the focused CMR protocol should be made depending on the quality of the information gained from TTE.

Box 1 Clinical questions in CMR assessment of MR A comprehensive cardiovascular magnetic resonance imaging (CMR) study should aim to answer the following clinical questions in the assessment of mitral regurgitation (MR) for consideration of mitral valvular intervention^3^.

**What is the aetiology of the MR?**

- Primary or secondary
- Presence and location of myocardial infarction on late gadolinium enhancement imaging

**How severe is the MR?**

**Are any signs present on imaging in asymptomatic patients that might indicate worse outcome if valve intervention is delayed?**

- Dilated left ventricle
- Borderline reduced left ventricular ejection fraction
- Dilated left atrium
- Progressive dilatation of the left ventricle and worsening of left ventricular function

**Has the MR worsened?**

- On longitudinal CMR studies, has the MR volume or MR fraction worsened?

### Cine images

**Fig. 2:**
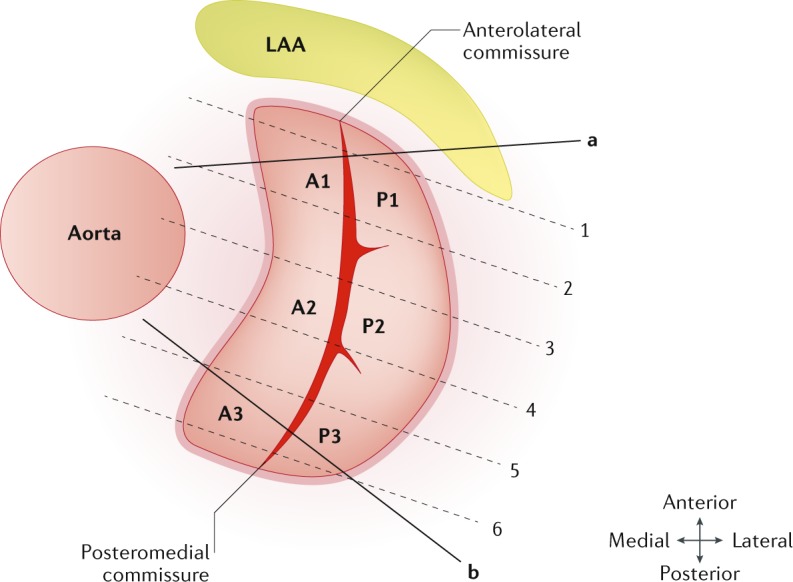
Mitral valve assessment planing during cine cardiovascular magnetic resonance imaging acquisition. A contiguous, long-axis, left ventricular outflow tract cine cardiovascular magnetic resonance imaging stack should be acquired to visualize and assess all the mitral valve cusps (A1–P1, A2–P2 and A3–P3). Extra planes to the commissural line also need to be considered, as demonstrated by the lines ‘a’ and ‘b’. LAA, left atrial appendage.

Standard cine CMR should be performed according to the SCMR recommendations^22^:

- Standard, long-axis, steady state, free-precession cine images: four-chamber (horizontal long-axis), two-chamber (vertical long-axis) and three-chamber (LV outflow tract view).
- A stack of contiguous cines perpendicular to the mitral commissures, transecting the principal line of coaptation, approximately in a modified LV outflow tract plane. These should have a slice thickness of 5 mm and no gap, with a temporal resolution of ≥45 ms (ref.^23^) (Fig. 2). The main aim is to cover all the mitral scallops: A1–P1, A2–P2 and A3–P3. Additional commissural cines are acquired perpendicular to the lines of coaptation next to each of the commissures if the commissures are at an oblique angle to the central coaptation line (Fig. 2, lines ‘a’ and ‘b’).
- Standard, contiguous, short-axis, LV cine stack with extended coverage of the mitral valve.
- Specific short-axis cine perpendicular to the tips of the mitral valve in systole (if an optimal image is not obtained within the LV stack).

#### Tips

On cine acquisitions, flow turbulence (for example, because of MR jets) produces spin–spin dephasing, which can be visualized as hypointense areas within the blood pool inside the relevant cardiac chamber. This phenomenon allows the observer to make a gross qualitative assessment of the MR. If steady state, free-precession cines do not show the flow void clearly, fast spoiled gradient-recalled echo cines with longer repetition and echo times can be performed to visualize the MR regurgitant flow void better. However, this qualitative MR assessment is very susceptible to changes in cine pulse sequences and therefore should be used cautiously. Visualization of the MR jet on cine images can, however, inform about the aetiology (in addition to cine imaging of leaflet anatomy and function) — eccentric jets associated with mitral valve leaflet prolapse versus a central jet associated with mitral annular dilatation or LV dilatation resulting in non-coaptation. Aetiologies of MR that are more challenging to diagnose, such as cleft mitral valve or perforated leaflets, can also be assessed well with a combination of cine and flow imaging. Owing to variations in breath holds, a stack of contiguous 5-mm slices can have spatial misalignment, which should be carefully judged during scanning. If the cusp views are missed during scanning, additional imaging can be required.

### Flow imaging

#### 2D phase-contrast flow

Currently, the standard approach to flow imaging is 2D phase-contrast, velocity-encoded imaging. For this approach, one-direction (through-plane), motion-encoded, cine gradient-echo sequences are used. The preferred sequence parameters for 2D phase-contrast imaging are included in the SCMR recommendations^22^:

- Aortic forward flow: a through-plane image plane should be placed at the sino-tubular junction in end diastole to quantify forward flow for the calculation of MR volume (LV stroke volume minus aortic forward flow). This plane should be perpendicular to the vessel. In published studies, baseline velocity encoding for aortic flow is 2.0–2.5 m/s. If there is significant aliasing, consider increasing the velocity encoding or imaging slightly higher than the previously prescribed phase-contrast through-plane.
- MR visual assessment: a long-axis LV outflow track phase-contrast stack that is perpendicular to the commissures, aligned with the direction of inflow and transecting the principal line of coaptation, is recommended to visualize MR jets in multiple planes. This visualization will clarify the aetiology of the MR. Alternatively, an experienced operator can plan a single image to capture through-plane flow on the atrial side of the valve.

#### Tips

Signal averaging can be used within the limits of breath-holding capabilities. Free breathing, respiratory navigator-based signal-averaging techniques can be applied to improve the temporal or spatial resolution if necessary. The potential for background flow offset errors can be reduced by ensuring that phase-contrast sequences are acquired with the region of interest (the ascending aorta) located at the iso-centre of the magnet to minimize any inhomogeneities in the magnetic field^24^. Background phase offset errors can significantly hinder the accuracy of flow measurement^25^, and background flow correction processes should be used, such as the interpolated automatic sequence^26^, where available. In patients with clinically significant aortic sinus turbulent flow, the through-plane image plane can be positioned at the level of the main pulmonary artery in the ascending aorta to quantify aortic forward flow.

In patients with arrhythmias (mostly atrial fibrillation), consider performing multiple phase-contrast acquisitions and also using arrhythmia-rejection sequences. If arrhythmia rejection is used for phase-contrast acquisitions, it should be similarly applied to the functional cine images. This approach will at least provide consistency between the flow and the functional measurements. Performing 2D phase-contrast through the mitral valve for forward and backward flow quantification is not recommended, mainly because this technique remains highly susceptible to through-plane mitral annular motion. Furthermore, for dynamic regurgitant jets, the acquisition plane cannot be adapted to the changing direction of flow.

#### 4D phase-contrast flow

The 2D phase-contrast can be swapped for 4D phase-contrast flow if local established expertise and technical knowledge exist to quantify transvalvular flow with this approach. 4D-flow CMR techniques offer further improvements in the assessment of MR and are entering clinical practice^27^. Advantages of MR quantification with the use of 4D-flow CMR include single-acquisition, single-sequence, retrospective analysis that allows valve tracking to account for motion throughout the cardiac cycle as well as direct measurement of MR^28^. Direct quantification of the regurgitant jet is particularly useful in pathologies involving multiple valves. A systematic review of 4D flow-derived methods for MR quantification identified seven studies that demonstrated that 4D flow-derived MR volume is similar to that derived using standard CMR methods and even to that derived using 3D transoesophageal echocardiography (TOE) methods^29^. In one study, a standard CMR method for quantification of MR volume and 4D flow-derived methods yielded similar results^30^.

For 4D-flow CMR, a retrospectively electrocardiogram-gated sequence covering the complete cardiac cycle, with a temporal resolution of ≥45 ms and a spatial resolution of 3 mm × 3 mm × 3 mm or higher is recommended^31^. The field of view should preferably cover the whole left ventricle, left atrium and aortic outflow track, including the proximal ascending aorta. Before analysis, 4D velocity data should be carefully checked for errors and, where possible, these errors should be resolved.

### LGE imaging

LGE imaging should be performed in accordance with published guidelines^22^. Contiguous, short-axis, LV stack LGE imaging is recommended, in addition to LGE in the three standard long-axis planes.

## Analysis

### Mitral valve anatomy

#### Leaflet morphology

A visual assessment should be made of all four components of the mitral valve: the anterior and posterior leaflets, the annulus, the subvalvular apparatus (papillary muscles), and LV contractility (including any regional wall-motion abnormalities). Abnormal leaflet morphology includes thickening, calcification, redundancy, perforation, vegetations, other masses and clefts. These abnormalities should be described in detail (diffuse versus focal, the size and the leaflet location). Abnormal subvalvular morphology can involve chordal rupture, thickening, fusion, very large vegetations and masses, which should similarly be described in detail by size and location. Abnormal annular morphology comprises dilatation and/or calcification (seen as signal loss). The long-axis stack is best for making the visual assessment of the mitral valve leaflets. Longitudinal mitral annulus disjunction distance is measured from the junction of the left atrial (LA) wall and the mitral valve leaflet to the top of the LV wall at end systole in long-axis cines and is defined as being clinically significant if the distance is ≥1.0 mm.

#### Leaflet motion

**Fig. 3:**
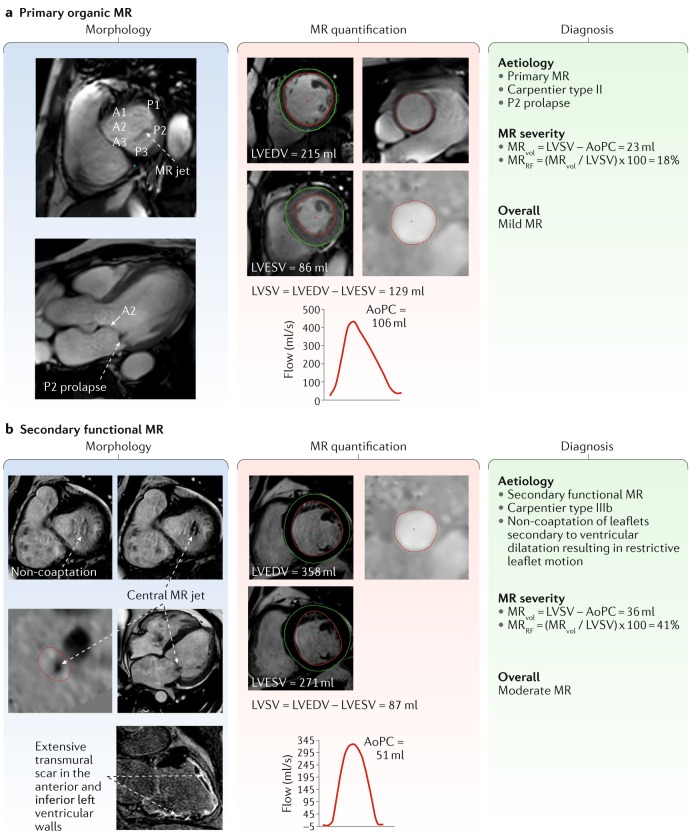
Case studies of primary and secondary MR. **a** | Mitral regurgitation (MR) assessment with cardiovascular magnetic resonance imaging in a patient with organic MR. Prolapse of the P2 can be seen on the three-chamber view during mid-systole (morphology panel, bottom image), and the resulting MR jet is visualized on the short-axis view (morphology panel, top image). The MR volume (MR_vol_) is quantified using the standard method: left ventricular stroke volume (LVSV) minus aortic phase-contrast forward volume (AoPC). **b** | MR assessment in a patient with ischaemic cardiomyopathy. Non-coaptation owing to ventricular dilatation is seen on the short-axis cines (morphology panel, top images). A through-plane phase-contrast acquisition shows the central MR jet (morphology panel, right-hand middle image). Late gadolinium enhancement imaging reveals extensive ischaemic myocardial scaring (Morphology panel, right-hand bottom image). LVEDV, left ventricular end-diastolic volume; LVESV, left ventricular end-systolic volume; MR_RF_, mitral regurgitation fraction.

Leaflet motion can be described using Carpentier’s classification: type I (normal leaflet motion); type II (excessive leaflet motion); and type III (restricted leaflet motion), subcategorized as type IIIa (restricted during both systole and diastole) and type IIIb (restricted only during systole). After a comprehensive review of leaflet morphology and motion, a possible aetiology for the MR should be described according to Table 2. Case studies are provided in Fig. 3. The aetiology should be consistent with the overarching diagnosis. Sometimes, a mixed picture of both primary and secondary MR can exist — for example, pre-existing secondary MR caused by dilated cardiomyopathy together with a newly torn chord or flail leaflet.

**Table 2:** Modified Carpentier’s classification of mitral valve morphology and MR aetiology

Type of leaflet motion	Normal mitral valve leaflet		Abnormal mitral valve leaflet	
	Leaflet lesion (morphology)	Aetiology: secondary MR	Leaflet lesion (morphology)	Aetiology: primary MR
Type I: normal leaflet motion	Annular dilatation	Dilated cardiomyopathy^a^ or left atrial dilatation	Leaflet perforation (tear)	Endocarditis
Type II: excess leaflet motion (prolapse or flail)	–	–	Elongation (rupture of chordae or papillary muscle)	Degenerative valve disease, endocarditis, trauma or ischaemic cardiomyopathy^a^
Type IIIa: restricted leaflet motion (both in diastole and systole)	–	–	Leaflet thickening (retraction), leaflet calcification, chordal thickening (retraction), fusion or commissural fusion	Rheumatic heart disease, carcinoid heart disease or dilated cardiomyopathy
Type IIIb: restricted leaflet motion (mainly in systole)	Left ventricular dilatation (aneurysm)	Ischaemic cardiomyopathy^a^ or dilated cardiomyopathy^a^	Papillary muscle displacement or chordae tethering	Ischaemic cardiomyopathy^a^ or dilated cardiomyopathy^a^

MR, mitral regurgitation. ^a^Mixed aetiology.

### Methods of MR quantification

Several qualitative and quantitative methods of MR assessment by CMR are available.

#### Qualitative assessment

The MR jet should be visualized using both cine and 2D phase-contrast CMR, as described in the MR assessment protocol. This approach is mainly performed by visual assessment of the MR jet on the basis of spin dephasing on cine images. MR jet characterization should include whether the jet is central or eccentric, and early, mid, late or pan systolic.

**Fig. 4:**
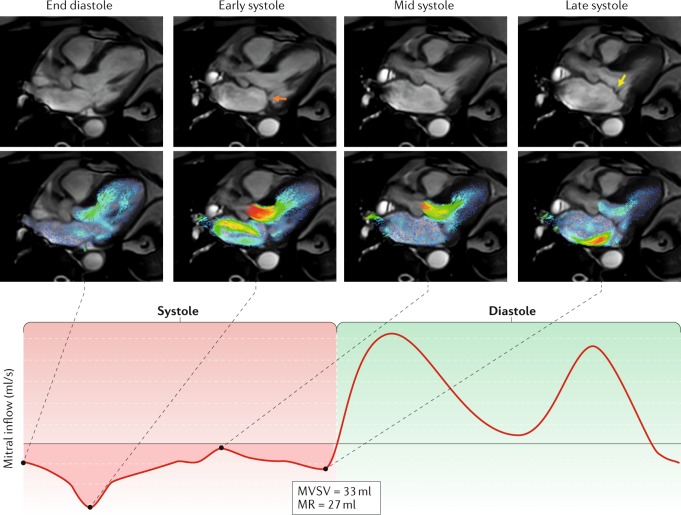
Time-resolved velocity vector visualization with CMR of dynamically changing MR jet. The top row shows cardiovascular magnetic resonance imaging (CMR) of three-chamber cines acquired during four phases of the cardiac cycle: end diastole, early systole, mid systole and late systole. The second row of images shows velocity vectors in the left atrium and the left ventricle superimposed on the three-chamber cines. These images allow the visualization of the A2–P2 scallops, demonstrating early systolic prolapse of the P2 scallop (orange arrow) resulting in a mitral regurgitation (MR) jet directed towards the medial interatrial septum, which settles in mid systole. A late-systolic, posteriorly directed MR jet (yellow arrow) can be appreciated as a result of A2 prolapse. The MR volume is quantified in the lower panel. This example highlights how cine CMR and augmented streamline visualization of the 4D-flow CMR can help to make a more dynamic pathophysiological diagnosis of the cause of MR. MVSV, mitral valve stroke volume.

4D-flow CMR allows for visualization of 2D velocity vectors in any plane, facilitating a comprehensive assessment of the blood flow dynamics in the left atrium^32,33^. MR jets are dynamic and can change directions during systole depending on mitral leaflet adaptations. Velocity vector visualization of LA flow coupled with cine CMR can help to understand the cause of the MR (Fig. 4). Velocity vector visualization of the velocity jet is preferred over spin dephasing because it provides truly quantitative, directional velocity data. In addition, this method can offer a better assessment of the extent of MR than is provided by Doppler imaging (which is single velocity-encoded imaging), especially when the MR jet is swirling within the left atrium.

#### Quantitative assessment

**Fig. 5:**
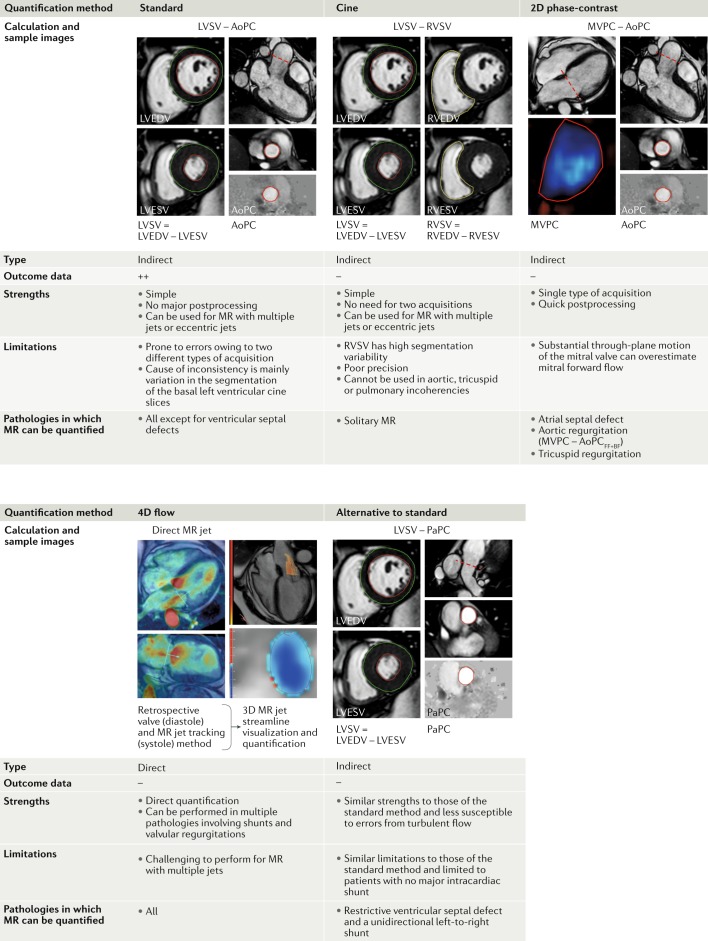
Main methods of MR quantification by cardiovascular magnetic resonance imaging. Prognostic and diagnostic outcome data are most available for the standard method of quantifying mitral regurgitation (MR) volume (MR_vol_), which is left ventricular stroke volume (LVSV) minus aortic phase-contrast forward volume (AoPC). Other methods have particular advantages or disadvantages. In routine clinical practice, cross-checking between methods is recommended. FF+BF, forward flow plus backward flow; LVEDV, left ventricular end-diastolic volume; LVESV, left ventricular end-systolic volume; MVPC, mitral valve phase-contrast stroke volume; PaPC, pulmonary artery phase-contrast stroke volume; RVEDV, right ventricular end-diastolic volume; RVESV, right ventricular end-systolic volume; RVSV, right ventricular stroke volume.

CMR planimetry of the anatomical mitral regurgitant lesion in patients with MR is feasible and allows quantification of MR, which has been shown to have good agreement with quantification by other invasive and noninvasive methods^34^. Quantification of mitral regurgitant volume and fraction is the recommended technique because most clinical outcome data are available. The MR volume can be obtained by four different methods (Fig. 5).

1. The difference between the LV stroke volume (LVSV) calculated using planimetry of cine steady state, free-precession images and the aortic (systolic) forward volume obtained by phase-contrast images (AoPC); the standard approach.
2. The difference between the LVSV and the RV stroke volume (RVSV) calculated using planimetry of cine steady state, free-precession images; this approach assumes no other valve regurgitation or haemodynamically significant shunt.
3. The difference between the mitral inflow stroke volume and the AoPC.
4. Direct quantification of MR flow by 4D-flow CMR, with retrospective mitral valve tracking.

Among these methods, the first is the most widely used and has the most prognostic data available^12,13^. This method allows the quantification of the regurgitant volume without considering regurgitant jet morphology. This approach is particularly helpful in patients with multiple or eccentric jets, or variable jets through systole. In addition, this approach is independent of the effects of aortic, tricuspid and pulmonary regurgitation. However, this method requires a combination of two acquisitions and is, therefore, subject to potential interscan variability. If any issues exist with the acquired AoPC to quantify MR by the LVSV – AoPC method, an alternative approach is to use the pulmonary artery flow (PaPC), as long as no intracardiac shunts exist (LVSV – PaPC). Moreover, the PaPC approach might be advantageous in certain circumstances because the pulmonary valve is less often diseased and therefore less susceptible to creating errors from turbulent flow. In patients with a restrictive ventricular septal defect and a unidirectional left-to-right shunt, the LVSV – PaPC method can be used to quantify MR. In patients with a bidirectional flow ventricular septal defect, this method is not applicable, and direct measurement of the MR jet should be considered. In patients with an atrial septal defect, the standard method (LVSV – AoPC) is still appropriate for quantification of MR and also allows the shunt to be assessed using the ratio of the pulmonary and aortic flows (PaPC:AoPC).

The difference in LVSV and RVSV can also be used to quantify MR^31^. However, given the relatively lower precision with which RVSV is quantified compared with LVSV, substantial bias in MR volume can be introduced between two operators, resulting in reduced reliability^35^. In addition, this method is not valid for patients with multiple valve lesions or shunt flow as a result of ventricular septal defects.

The third method is, in theory, valid for patients with multiple valve lesions or shunt flow but, in practice, this method often has substantial errors. 2D phase-contrast CMR requires static imaging planes that cannot adapt to through-plane valve motion or the changing location of the mitral valve and the changing direction of inflow or regurgitant jets^36,37^. This method is also susceptible to measuring entrained blood already in the left atrium as part of the regurgitant jet if the imaging slice is too far from the orifice or the region of interest is too large. Furthermore, this method requires two acquisitions, which can be subject to variability.

**Fig. 6:**
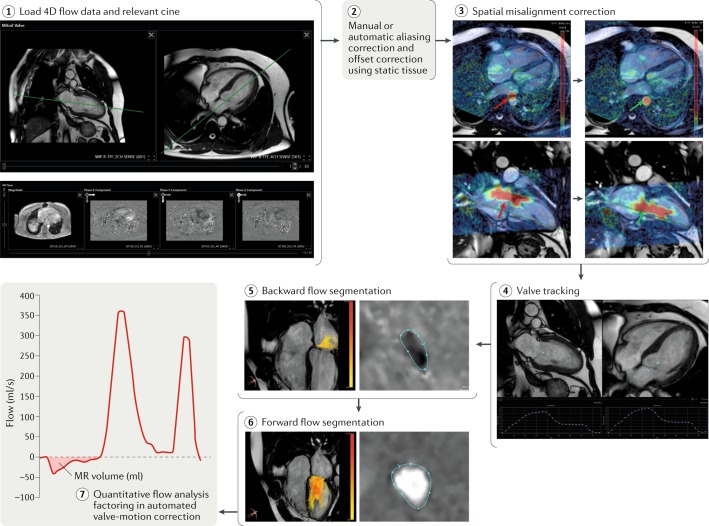
4D-flow cardiovascular magnetic resonance imaging analysis. Step 1: load the two-chamber and four-chamber cardiovascular magnetic resonance imaging cines (upper panel) and 4D phase-contrast flow and 3D phase-contrast data (lower panel). Step 2: depending on the software tool, perform manual or automated aliasing and velocity offset corrections with the use of the static background tissue. Step 3: an attempt to check for spatial misalignment between the cines and the 4D-flow data should be made because the presence of any major misalignment between them will introduce errors in the quantification of valvular flow. The red arrows indicate the spatial misalignment of both the descending aortic flow (in systole) and the mitral inflow (in diastole). The green arrows indicate how this misalignment has been corrected. Step 4: on both the two-chamber and the four-chamber cines, place two landmark points to mark the valve plane. This marking should be done for the complete cardiac cycle. Software solutions (for example, CAAS software, PIE Medical Imaging, Netherlands) can now automatically track the valve over the complete cardiac cycle. After ensuring the valve is properly tracked, generate a phase-contrast, valvular reformatted plane. Steps 5–6: on the valvular reformatted plane, segment the mitral regurgitant backward flow during left ventricular systole and the forward flow during diastole. The regurgitant plane should be perpendicular to the regurgitant jet. If multiple jets exist, one should make an attempt to evaluate each of them to quantify the total mitral regurgitation (MR). Step 7: quantify valvular forward and backward flow after accounting for the through-plane motion of the valve plane. Similar steps can be used to quantify aortic, tricuspid and pulmonary valvular flows with the use of the 4D-flow data set.

The 4D-flow CMR, retrospective valve-tracking method (Fig. 6) produces a direct quantification of MR by quantifying flow directly at the valve and is valid in the presence of multiple valve lesions or shunt flow^38^. This approach overcomes the limitations of the third method described above, but acquisition times and postprocessing can be challenging^27^. In retrospective valve tracking, a dynamic reformatted 2D phase-contrast plane is reconstructed by tracking the mitral annulus over the whole cardiac cycle^28,32,38^. MR jets are quantified by defining a systolic reformatted plane perpendicular to the single jet or individually for multiple jets. Alternatively, if the MR jet is too complex, a reconstructed aortic plane using the retrospective valve-tracking method can be used to quantify AoPC. This measurement can then be used to quantify MR volume or fraction using the standard LVSV – AoPC method.

Finally, in routine practice, cross-checking MR quantification between methods is useful to reduce uncertainty. Particularly in isolated MR, LVSV – AoPC can easily be cross-checked with LVSV – RVSV or even LVSV – PaPC.

### Volumetric cine analysis

#### LV and RV volume assessment

LV and RV volume quantification is performed according to standard methods^33^. The use of analysis software that allows demarcation of the base of the ventricles on the long-axis images is recommended. LV end-diastolic volume and LVSV are used for the standard method of MR volume calculation (see below) as well as for determining the degree of LV dilatation in response to MR.

#### LA assessment

LA volumes should be assessed using the biplane area–length method^39–41^. On the basis of the cine long-axis four-chamber and two-chamber views, the contours of the endocardial borders are delineated at end systole (LA diastole). The LA appendage should be included in the atrial volume, but the pulmonary veins should be excluded. 3D volume methods on the basis of short-axis stacks can be performed and are more accurate than the 2D biplane method, but both the acquisition and postprocessing are much more time consuming.

## CMR reporting of MR assessment

A CMR report for MR assessment should include the standard reporting details described in the CMR standards (Box 2). The report should include a detailed description of morphological and/or functional characteristics of the mitral leaflets, annulus and chordae tendineae. In addition, a description of MR jet characteristics (such as central or eccentric; single or multiple; early, mid, late or pan systolic) and expansion in the left atrium should be included. The CMR report should mention the method of MR quantification. If any nonstandard method is chosen, a clear reason why it was adopted should be detailed in the report. In the report conclusion, adding the morphological and functional correlates to the aetiology of the MR, including primary or secondary MR and Carpentier’s functional class of MR, is helpful.

Box 2 CMR report for MR assessment **Include information on the indication for cardiovascular magnetic resonance imaging (CMR)**

**CMR protocol used for mitral regurgitation (MR) assessment:**

- Noncontrast MR assessment
- Contrast MR assessment
- Heart rate and blood pressure

**Standard CMR report, including details on:**

- Left ventricular and right ventricular regional and global function
- Presence of scarring or infarction on late gadolinium enhancement imaging, with description of myocardial viability and left atrial (LA) and right atrial size
- Any other pathology identified should be described

**Mitral valve qualitative assessment**

- A detailed description of mitral valve characteristics, including:
  - Leaflets: thickened (base, mid or tips), calcification, restricted motion, tethered, bowing, prolapse or flail
  - Annulus: annular calcification, fibrosis on late gadolinium enhancement imaging, mitral annular disjunction >1 mm
  - Chordae: thickened, short, restrictive, rupture or tear
- A description of MR jet characteristics:
  - Central or eccentric MR jet
  - Single or multiple MR jets
  - Early, mid, late or pan systolic
  - MR jet expansion in the left atrium

**Quantitative analysis of complete CMR study**

- Dimensions, mass (corrected for body surface area) and function
  - Left ventricle: end-diastolic volume, end-systolic volume, stroke volume, ejection fraction and mass
  - Right ventricle: end-diastolic volume, end-systolic volume, stroke volume and ejection fraction
- MR (method used to quantify)
  - MR volume (ml)
  - Regurgitation fraction (%)
- LA size
  - LA volume (ml)
  - LA annulus diameter (cm)

**Final report conclusions**

- Morphological diagnosis of the aetiology of MR (primary or secondary) and/or Carpentier’s functional class of MR
- Degree of MR
- Degree of LA dilatation
- Left ventricular function and degree of dilatation
- Presence, location and degree of myocardial scar or replacement fibrosis

## Reference values to grade MR

Owing to the lack of a true gold standard, which method quantifies MR severity with the highest degree of accuracy and reliability is unknown. Nonetheless, prognostic studies that demonstrate the superiority of CMR quantification of MR can guide clinical decision-making. Echocardiographic quantification of MR generally shows a bias towards much higher regurgitant volumes than those measured by CMR, so the thresholds that define severity might need to differ according to the imaging technique used. Table 3 details the methods and CMR grading used in the three most relevant publications so far. If other methods are used to quantify MR volume or regurgitation fraction, such as 4D-flow CMR, similar thresholds could be used. However, future large studies are needed to compare different MR quantification methods directly with outcomes to clarify the applicability of the thresholds for different methods.

**Table 3:** Recommended grading of MR by CMR assessment

Type of MR	Grading of severity			
	Mild	Moderate	Severe	Very severe
Primary	MR_RF_ <20%^a^	MR_RF_ = 20–39%^a^	MR_RF_ 40–50%; MR_vol_ >55–60 ml	MR_RF_ >50%
Secondary	MR_vol_ <30 ml	MR_vol_ = 30–60 ml	MR_vol_ ≥60 ml	–

From refs^12,13,19^. CMR, cardiovascular magnetic resonance imaging; MR, mitral regurgitation; MR_RF_, mitral regurgitation fraction; MR_vol_, mitral regurgitation volume. ^a^Not study-based; mainly expert opinion.

## CMR in clinical pathways

In routine clinical pathways, if MR is suspected on the basis of clinical signs and symptoms, TTE assessment of MR can determine its aetiology, assess its severity and measure the haemodynamic consequences on the left ventricle. In patients in whom the degree of MR is uncertain, especially between moderate and severe MR, further tests are considered. TOE has been the second-line imaging test not only for clarification of the aetiology but also for assessing the degree of MR. TOE has higher spatial and temporal resolution than either TTE or CMR. However, TOE remains a semi-noninvasive test and relies on successful oesophageal intubation. Although TOE is widely available and is less expensive than CMR, risks are associated with a TOE examination^42^. Overall, with the advent of CMR methods for MR assessment, the real value of TOE will be in providing high-resolution 2D and 3D dynamic imaging of the mitral apparatus, mitral valve and scallops to inform and plan surgical intervention, in guiding mitral valve percutaneous interventional procedures and, finally, for intraoperative checks before and after mitral valve intervention. Importantly, MR volume quantified by 3D TOE has a high level of agreement with that obtained by standard CMR methods^43^.

In patients in whom further assessment of MR severity is needed, we recommend CMR as a second-line noninvasive test. CMR is also recommended in asymptomatic patients with severe MR for further clarification of LV and LA volumes. If a clinical decision is made to ‘wait and watch’, the focused CMR study proposed in this Consensus Statement can be used to perform longitudinal volumetric and flow assessment to investigate any progression of the MR and associated volume overload on the left ventricle. CMR is not currently recommended for further investigation of vegetation in patients with suspected infective endocarditis-associated MR. This lack of recommendation is mainly because the current spatial resolution of CMR is not high enough, and CMR generates averaged time-resolved images over several cardiac cycles rather than live images. During multidisciplinary meetings involving imaging cardiologist–radiologists, nonimaging cardiologists and surgeons, we recommend actively discussing all imaging options, including CMR-derived MR metrics, to encourage wider familiarity with CMR methods. In summary, TTE, TOE and CMR are likely to provide complementary information to guide treatment and surveillance in patients with MR.

## Future directions

The evidence that CMR can be used to quantify MR accurately and to predict outcomes^12,13^ makes CMR quantification of MR an attractive tool for future use in randomized controlled trials. The benefit of mitral valve intervention on the basis of the severity of MR (by any modality) has never been studied in a randomized controlled trial, even though mitral valve repair is considered acceptable for asymptomatic patients with a repairable mitral valve according to both European and US guidelines^3,5^.

Future clinical studies are also needed to address whether theoretically more accurate methods of directly quantifying MR, such as retrospective valve tracking using 4D-flow CMR data, are superior to the currently established methods. A need also exists for widespread adoption of robust background flow offset correction methods to provide MR practitioners with confidence in flow quantification. The inaccuracy of quantification in some patients and on some systems is a major barrier to the use of CMR both in MR and in valve disease more generally. Good CMR thresholds for defining the severity of MR are also required, ideally on the basis of outcome data. Existing quantitative thresholds borrowed from echocardiographic data are unsuitable, with wide variation between echocardiography and CMR^12,13^.

## Conclusions

The assessment of MR by CMR has great utility. CMR is a robust clinical imaging test for the comprehensive assessment of mitral valve morphology and the quantification of MR, with high levels of accuracy. Evidence suggests that CMR can be used to guide and inform clinical outcomes and prognosis in patients with MR. Emerging methods, including 4D-flow CMR, show great promise to improve the precision and accuracy of MR quantification. However, further studies to investigate the clinical benefit of 4D-flow CMR are warranted.
